# Near-infrared indocyanine green angiography in recognizing bowel ischemia in emergency surgery: game changer or overrated?

**DOI:** 10.1515/iss-2024-0013

**Published:** 2024-07-15

**Authors:** Anastasia Christofi, Thilo Traska, Dimitrios Dimitroulis

**Affiliations:** Department of General, Visceral and Vascular Surgery, 40556 Agaplesion Bethesda Hospital Wuppertal, Wuppertal, Germany; Hellenic Minimally Invasive and Robotic Surgery (MIRS) Study Group, Athens Medical School, National and Kapodistrian University of Athens, Athens, Greece; Second Department of Propedeutic Surgery, Laiko Hospital, Athens, Greece

**Keywords:** bowel ischemia, emergency, fluorescence angiography, indocyanine green, surgery

## Abstract

Assessing bowel perfusion in emergency intestinal surgery can prove challenging even for experienced surgeons. The necessity of a technological tool assisting clinicians is undisputed. Near-infrared indocyanine green (NIR-ICG) angiography has been increasingly used in elective colorectal surgery to evaluate intestinal perfusion with promising results. This review aims to answer whether a similar outcome can be observed in acute cases of bowel ischemia. We conducted online research of the literature using keywords such as “indocyanine green”, “bowel”, “emergency” and “ischemia”, to identify articles concerning the use of ICG-angiography in evaluating bowel perfusion during emergency operations. PubMed was the primary database. 11 articles were included in this systematic review with a total of 358 patients. Most papers showed a positive effect after using NIR-ICG-angiography, whereas one study indicated the limitations of the method by exhibiting increased reoperation and mortality rates. Moreover, a significant variation in indocyanine green (ICG) dose and fluorescence identification systems was observed. NIR-ICG-angiography has the potential to become a fundamental tool in emergency intestinal operations. Nevertheless, additional research, especially high-quality, randomized studies, as well as quantification techniques are still needed to support these preliminary observations.

## Introduction

Identifying the extent of bowel ischemia, whether caused by mesenteric occlusion, ileus, or incarcerated hernia, is a critical skill in emergency gastrointestinal surgery. Removing insufficient length of the bowel can lead to reduced perfusion of the anastomosis with consequent anastomotic leakage and the plethora of downsides correlated with it (reoperation, increased morbidity and mortality, increased hospital stay). On the other hand, extensive bowel resection, when not necessary, may increase operation time and lead to conditions like short bowel syndrome or malabsorption [1].

Especially for younger surgeons, recognizing the correct length of the ischemic bowel can prove to be a challenge. A variety of methods regarding the evaluation of intestinal viability intraoperatively has been described [2, 3]. Traditionally, the evaluation is based on the clinical assessment of the surgeon. The main factors helping with the decision are the color of the bowel serosa, the presence of peristalsis, the presence of pulsation, as well as bleeding of marginal arteries. Not only is this method subjective and based on the surgical team’s experience, but multiple factors may also cause misinterpretation. Vasoconstriction caused by hypotension, vasospasm, or noradrenergic drugs can temporarily affect the presence of pulsation in the distal mesenteric area. Moreover, peristalsis can be present even in ischemic bowels, whereas color changes of the bowel don’t necessarily correlate to the length of ischemia [4, 5]. In conclusion, Kaliczek et al. showed that the surgeons’ clinical assessment regarding the risk of anastomotic leakage lacks predictive accuracy [6].

It is obvious that more objective and reliable diagnostic tools for the evaluation of bowel perfusion are needed. A technique that fulfills these criteria and is currently highly discussed is the near-infrared indocyanine green (NIR-ICG) fluorescence angiography [7], [8], [9].

Indocyanine green (ICG) was first developed as a photographic dye for near-infrared photography in 1955 by Kodak research laboratories [9]. It received FDA approval in 1959 and was initially used in a clinical setting for the assessment of cardiac output and hepatic function, as well as for retinal angiography [9]. ICG has a peak spectral absorption at 800 nm. Once injected intravenously it rapidly bounds to plasma protein, so after being excited to fluorescence by an infrared light source it can accentuate vascular flow in real time. It is subsequently taken up almost exclusively by the liver and secreted in the bile with a half-life of 2–4 min.

In the previous decades, we have seen an increased interest in the use of ICG in surgery, as evidenced by the considerable increase in the volume of ICG-related publications [9]. The largest share of studies concerns the evaluation of anastomosis in colorectal surgery. Newer studies have moved this method to the upper gastrointestinal tract and the assessment of gastric perfusion in the setting of gastroesophageal reconstruction. Other applications include the detection of gastrointestinal sentinel nodes, bile duct imaging as well as intraoperative ureter identification to prevent injury [8, 9].

Since the use of NIR-ICG fluorescence for perfusion assessment in intestinal surgery seems to be increasingly prevalent, the question arises if this technique can show benefits applied in a similar setting in emergency surgery. The purpose of this scoping review is to collect the bibliography necessary to answer the question of whether intraoperative ICG fluorescence angiography can help in decision-making regarding the extent of bowel ischemia and thereafter bowel resection in emergencies. Some preclinical studies on animal models already exist [10, 11], but investigations in clinical settings are crucial in answering this question.

## Materials and methods

**Figure 1:**
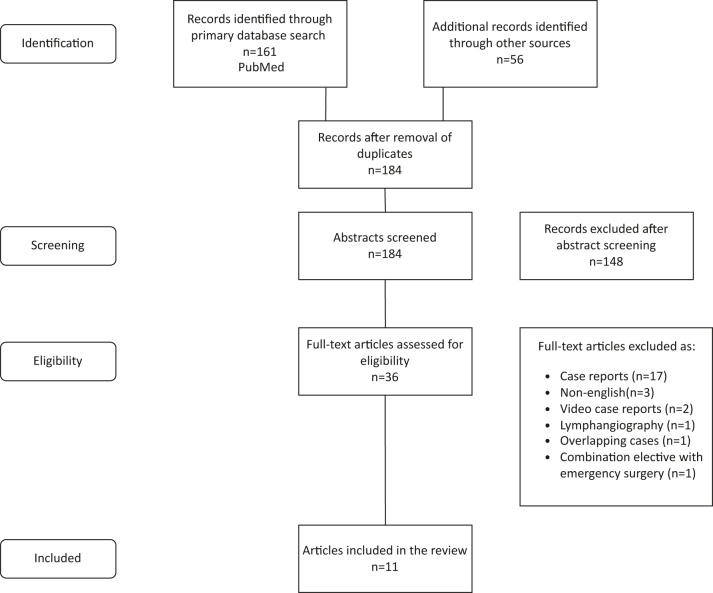
PRISMA flow diagram.

For the purpose of this scoping review, we used the PRISMA extension for scoping reviews, since a meta-analysis was not possible with the available data [12]. We conducted a literature search in PubMed as the primary database. Moreover, we used additional sources such as ResearchGate and Cochrane Library to yield more results. Specific terms regarding the use of ICG fluorescence angiography in emergency abdominal surgery were used. More specifically the search terms used in PubMed were “indocyanine green” AND/OR “indocyanine green fluorescence” AND/OR “fluorescence” AND (“colorectal” OR “rectal” OR “colon” OR “bowel”) AND (“ischemia” or “emergency”). The final search was conducted on the 15th of January 2024. Two authors independently performed the literature screening, with disagreements being resolved through the third author. After identifying eligible studies, the snowball method was utilised through a manual search of the references to help identify further relevant papers for screening. Figure 1 presents the PRISMA flow diagram of the article identification, screening, and elimination process.

### Inclusion criteria

Full-text articles in the English language, concerning the use of NIR-ICG fluorescence angiography to evaluate bowel perfusion in gastrointestinal surgery in the course of emergency operations were included.

### Exclusion criteria

Non-English papers, studies regarding elective colorectal or other types of operations, studies in paediatric populations, and articles relating to the use of ICG outside angiography (e.g. lymphography) were excluded.

### Data extraction

Our search in PubMed resulted in a total of 161 articles related to the use of NIR-ICG angiography in emergency abdominal surgery. 56 additional related articles were identified through various other sources, for a total of 217. After removing duplicates and screening abstracts we were left with 36 articles. 25 of these full-text articles were excluded for various reasons (17 case reports, three non-English full-text studies, two being video case reports, one concerning a combination of elective and emergency operations, one describing the use of ICG in fluorescence lymphoscintigraphy, and 1 case of overlapping patients reported, where the most recent article was chosen). We were left with a total of 11 articles to include in the scoping review, four single-center retrospective reviews, three single-center prospective studies, two single-center retrospective analyses of prospectively collected data, one multi-center retrospective analysis, and one technical note.

## Results

After adding the number of patients included in all studies, we came up with a total of 358 patients. The included articles reported a number of different pathologies that led to emergency operations. A total of three papers (Ryu [13], Nakashima [14], Guerra [15]) reported on the use of NIR-ICG angiography in cases of strangulated bowel obstruction. Two studies (Osterkamp [16] and Afifi [17]) involved cases of emergency operations as a result of abdominal trauma. The analysis of Karampinis [18] concerns the use of ICG in patients with acute mesenteric ischemia, whereas Ishizuka [19] focused on non-occlusive mesenteric ischemia. Ganguly [20] reported on two cases of bowel ischemia due to incarcerated hernias. Lastly, three papers (Liot [1], Joosten [21] and Osseis [22]) included a multitude of different causes leading to emergency surgery. Table 1 summarizes the main characteristics of the studies included in this review.

**Table 1:** The main characteristics of the studies included in the review.

Author	Year of publication	Type of study	Indication for surgery	Patients enrolled
Joosten et al. [21]	2022	Multi-center retrospective analysis	Varied	n=93
Guerra et al. [15]	2021	Technical note, single-center study	Strangulated bowel obstruction	n=71
Liot et al. [1]	2018	Single-center retrospective study of prospectively collected data	Varied	n=56
Karampinis et al. [18]	2018	Single-center retrospective analysis of prospectively collected database	Acute mesenteric ischemia	n=52
Ryu et al. [13]	2021	Single-center retrospective review	Strangulated bowel obstruction	n=38
Osterkamp et al. [16]	2021	Single-center prospective study	Abdominal trauma	n=20
Nakashima et al. [14]	2021	Single-center retrospective study	Strangulated bowel obstruction	n=14
Ishizuka et al. [19]	2015	Single-center prospective analysis	Non-occlusive mesenteric ischemia	n=6
Osseis et al. [22]	2018	Single-center retrospective study	Varied	n=3
Afifi et al. [17]	2021	Single-center retrospective review	Abdominal trauma	n=3
Ganguly et al. [20]	2021	Single-center prospective series	Incarcerated hernia	n=2

The largest study (Joosten [21]), a multi-center, retrospective case series, included 93 patients undergoing emergency operations as a result of bowel ischemia. The use of ICG-angiography resulted in a change in surgical management (CoM-FA) in 27 patients (29 %). A more aggressive strategy, resulting in more extended bowel resection, was observed in six patients (6 % overall). No re-operations were necessary for this group. In 21 cases (22 %) the use of ICG led to a more conservative approach with a median bowel length of 50 cm preserved. On four of those patients, no resection was performed. The re-operation rate of this group, both planned and unplanned, was 29 % (6 patients), with three patients presenting further ischemia. In comparison, the group without a modification of surgical course because of ICG (No CoM-FA) had a re-operation rate of 8 % (5 patients), one of which exhibited progressive ischemia. Moreover, a higher mortality rate was observed in the CoM-FA group (24 % in the more conservative and 33 % in the more aggressive strategy) than the No CoM-FA group (15 %). The authors of the article attribute this to a generally higher ASA score and a higher percentage of mesenteric ischemia in the patients of the CoM-FA group. Regarding the higher reoperation and advancing bowel ischemia in the CoM-FA group, the authors raised concerns about the objectivity of the method, being carried out by multiple surgeons of different expertise levels and different experience with ICG. Overall, they see positive results with the use of ICG-angiography in the acute setting, emphasizing however the need for more prospective studies, surgeon training, and standardizing as well as quantifying the method.

The two consequent biggest studies in sample size, Liot [1] and Karampinis [18], with 56 and 52 patients enrolled respectively, focused on the question of whether NIR-ICG-angiography provides additional information intraoperatively compared to macroscopic evaluation, leading to a change in the surgeons’ decision-making. Furthermore, the postoperative outcomes, including re-operation rates and mortality were examined. Liot [1] reported that in 33 % (n=18) of the cases, the operative strategy was altered following the results of the ICG-angiography. Out of these, in 67 % (n=12) a more conservative approach was selected than initially planned, thus avoiding unnecessary resection of segments of the gastrointestinal tract. In the rest 33 % (n=6) of the altered strategy cases the surgeon performed either a resection where none was planned or the resection of a larger segment. A total of 16.1 % of the overall patients required reoperation, though almost half of which were a planned second look. A total of 10 % of the total 20 anastomoses performed (n=2) developed anastomotic leakage, both of them in the group where the planned operative strategy was not changed after the use of NIR-IGC-angiography.

Karampinis [18] reported 34.6 % (n=18) of cases where a disagreement between the macroscopic assessment of the intestinal perfusion and the results of NIR-ICG-angiography occurred. The study identified 6 cases in which the dissonance of the two methods potentially led to a benefit for the patient. Out of the 6, in 5 cases a smaller part of the bowel was resected or no resection was performed, whereas in the final case a segment of the bowel, that was macroscopically evaluated as well perfused, showed a malperfusion in the NIR-ICG angiography. All the patients recovered without further resections needed. 10 more cases were grouped in the article as cases without a clinical benefit from the discrepancy between NIR-ICG angiography and clinical evaluation. Nine patients died in the early postoperative period of causes not related to the operation, however, no certain errors in the NIR-ICG evaluation were detected. In 1 case the NIR-ICG angiography showed signs of inadequate perfusion, whereas macroscopically the affected bowel segment seemed vital. No resection was performed and the patient recovered without complications, making the findings of the NIR-ICG-angiography false positive for bowel ischemia. In further two cases, NIR-ICG-angiography gave a false negative result; the histological results in the first case and intraoperative endoscopy in the second case showed mucosal ischemia, while ICG-angiography evaluated the perfusion as normal.

Ryu [13] compared the use of NIR-ICG-angiography during laparoscopic surgery for strangulated bowel obstruction vs. palpation in open surgery as evaluation tools to determine bowel viability in a total of 38 patients. The ICG group consisted of 16 patients (42 %), whereas the non-ICG group included 22 patients (58 %). The rate of bowel resection was significantly lower in the group where NIR-ICG-angiography was performed (25 %) in comparison to the non-ICG group (50 %). Necrotic tissue findings were higher in the ICG-group, thus revealing a higher accuracy of the technique. Furthermore, the rate of postoperative complications in the ICG group was also significantly lower than in the non-ICG group (6.3 % vs. 40.9 %), therefore demonstrating NIR-ICG-angiography as a reliable method for evaluating intestinal blood flow in laparoscopic emergency settings.

Even though Guerra [15] included 71 patients with small bowel obstruction in his report, the use of NIR-ICG-angiography only came into consideration for 16 patients (23 %). The remaining 55 (77 %), exhibited obvious macroscopical signs of either ischemia or good bowel perfusion, making the use of NIR-ICG-angiography redundant. Out of the aforementioned 16 patients, ICG was used in 7 (Group A – 44 %), while in the remaining 9 (Group B – 56 %), bowel viability was decided through the surgeon’s clinical observation. Both groups presented similar results regarding postoperative morbidity, conversion to laparotomy, resection or preservation of bowel, and length of hospital stay. In conclusion, NIR-ICG-angiography was characterized as a safe and easily reproducible tool in the laparoscopic treatment of bowel obstruction.

Nakashima’s [14] cohort study of 14 patients with strangulated bowel obstruction combined the use of NIR-ICG-angiography with macroscopic findings for the decision-making regarding surgical strategy. Bowel resection was performed in 4 cases (29 %) and the results of microscopic examination confirmed bowel necrosis in all of these cases. In the rest 10 cases (71 %) no bowel was resected. Except for one case of aspiration pneumonia, no postoperative complications were observed. The results of this study coincide with the previous ones in confirming the usefulness and reliability of ICG in the laparoscopic treatment of strangulated bowel.

Regarding strangulated bowel, Ganguly [20] reported 2 cases of incarcerated hernias, where ICG findings contradicted the macroscopic evaluation of bowel viability. Although the bowel segments seemed ischemic to the naked eye, ICG demonstrated perfusion and the bowel was preserved. No complications were presented postoperatively.

Ishizuka [19] focused his paper on cases of non-occlusive mesenteric ischemia. He described four out of six patients (67 %) where dissonance between NIR-ICG-angiography and macroscopical evaluation was observed. All four patients underwent a more extensive bowel resection, following the results of the NIR-ICG-angiography. The histological reports supported the findings of the NIR-ICG-angiography and all patients recovered well, except one who died of causes unrelated to the operation.

Osseis [22], described a case of bowel ischemia as a result of a strangulated femoral hernia. Macroscopically, after warm irrigation, the bowel perfusion seemed improved, NIR-ICG-angiography showed however poor perfusion. No resection was performed. In the further course, bowel perforation due to ischemia occurred, making a relaparotomy with resection necessary. In the same report, ICG and macroscopical findings agreed in the next case, leading to successful preservation of bowel. In the third case of the report, ICG findings lead to saving a part of the ileum, which seemed affected by ischemia macroscopically.

Lastly, the two papers reporting on the use of NIR-ICG-angiography in the acute setting of abdominal trauma (Osterkamp [16] and Afifi [17]), reviewed a group of 20 and three patients respectively. In the study of Osterkamp [16] the operation course was modified in 70 % of the cases (14/20) due to the results of the NIR-ICG-angiography. A major change in surgical decisions was observed in 30 % (6/20). The postoperative complications that occurred were unrelated to surgical management, showing successful use of the NIR-ICG-angiography. Furthermore, Afifi’s [17] case series demonstrated one more instance where NIR-ICG-angiography correctly revealed ischemic visceral tissue that appeared perfused macroscopically and 2 cases where NIR-ICG-angiography confirmed the macroscopical evaluation of bowel perfusion.

Summarized, out of a total of 358 patients enrolled in the above studies, NIR-ICG-angiography was used in 272 cases. The aetiology of bowel ischemia and the number of cases where NIR-ICG angiography was used are shown on Table 2. In 86 cases a dissonance between the macroscopical evaluation and ICG findings was noted. In most cases the use of ICG allowed for a more conservative surgical approach and bowel preservation, there were however cases where a more aggressive approach ensued. Even though most articles reported positive results, the largest one [21] showed increased reoperation, progressive tissue ischemia, and mortality rates in the group acting according to the ICG findings. Moreover, Karampinis [18] showcased two instances of false negative results with the NIR-ICG-angiography, where good bowel perfusion was shown, even though ischemia was consequently detected endoscopically and histologically.

**Table 2:** Aetiologies of bowel ischemia and cases where NIR-ICG-angiography was used.

Aetiology of ischemia	No. of patients	No. of patients where ICG was used
Strangulated bowel obstruction	183	97
Mesenteric ischemia	101	101
Abdominal trauma	23	23
Incarcerated hernia	8	8
Nonocclusive mesenteric ischemia (NOMI)	6	6
Volvulus	4	4
Other (including perforation, occlusive tumour, diverticulitis)	33	33

A big heterogeneity was observed regarding the device used for detecting the ICG fluorescence, with some studies using more than one system. Pinpoint endoscopic fluorescence imaging from the company Stryker was used in three studies for laparoscopic operations, whereas SPY Portable Handheld Imager (SPY-PHI) Elite Imaging System, also from Stryker, was utilized in three of the articles for open surgery cases. Two studies were carried out with the help of PDE (Photodynamic Eye) of Hamamatsu Photonics (open surgeries) while the systems IMAGE1 S™ OPAL1 of Karl Storz, VISERA ELITE 2, system of Olympus and 1688 AIM 4K of Stryker were used each twice. Lastly, one article reported using 1588 AIM 4K, Stryker.

Similarly heterogeneous was the dose of ICG dye reportedly administered in the different articles. The total dose of the ICG given was mostly specified in milligrams but was sometimes disclosed in milligrams per kilogram of body weight. That being said, most studies used either a total of 5 mg (3/11 studies) or 7.5 mg (2/11 studies), with Ryu [13] describing ICG injection doses of 5 mg for patients weighing under 75 kg and 7.5 mg for patients over 75 kg. Dosages of 2.5 mg [19], 12.5 mg [22], and 25 mg [15] were administered each in one study. Afifi [17] reported a dose of 0.25 mg/kg whereas in the study of Joosten [21] no ICG dosage was mentioned. Table 3 presents a summary of the ICG doses injected as well as the fluorescence imaging system used.

**Table 3:** Fluorescence imaging system and ICG dose.

Study	Fluorescence imaging system (manufacturer, country)	ICG dosage
Joosten et al. [21]	– PINPOINT (Stryker, USA)	Not stated
	– 1688 AIM 4K (Stryker, USA)	
	– SPY-PHI Elite Imaging System (Stryker, USA)	
Guerra et al. [15]	IMAGE1 S™ OPAL1 (Karl Storz, Germany)	25 mg in 5 mg boluses
Liot et al. [1]	PINPOINT (then Novadaq, Canada, now Stryker, USA)	7.5 mg
Karampinis et al. [18]	– PINPOINT (Novadaq, Canada, now Stryker, USA)	7.5 mg
	– SPY-PHI Elite Imaging System (Novadaq, Canada, now Stryker, USA)	
Ryu et al. [13]	– VISERA ELITE II (Olympus, Japan)	5 mg <75 kg of BW/7.5 mg >75 kg of BW
	– 1688 AIM 4K (Stryker, Japan)	
Osterkamp et al. [16]	SPY-PHI Elite Imaging System (Stryker, USA)	5 mg
Nakashima et al. [14]	VISERA ELITE II (Olympus, Japan)	5 mg
Ishizuka et al. [19]	Photo Dynamic Eye (Hamamatsu Photonics, Japan)	2.5 mg
Osseis et al. [22]	Photo Dynamic Eye (Hamamatsu Photonics, Japan)	12.5 mg
Afifi et al. [17]	IMAGE1 S™ OPAL1 (Karl Storz, Germany)	0.25 mg/kg of BW
Ganguly et al. [20]	1588 AIM 4K (Stryker, USA)	5 mg

ICG, indocyanine green; BW, body weight.

### Discussion

The need for a new, reliable method to evaluate tissue perfusion in intestinal surgery has become apparent through the increasing number of studies concerning the use of NIR-ICG-angiography for this purpose. Several systematic reviews and metanalyses have been published in recent years, discussing the efficacy of NIR-ICG-angiography in reducing anastomotic leakage in elective colorectal surgery [23], [24], [25], [26]. The results are encouraging, even though in most cases the necessity for further high-quality randomized studies and standardization of the method are highlighted.

In accordance with the aforementioned studies, the papers we were able to include in our review are for the most part single-center, retrospective studies. This only accentuates the need for prospective, randomized controlled trials regarding the use of NIR-ICG-angiography in cases of acute bowel ischemia.

Most studies included in our review were able to showcase a benefit, or at least non-inferiority of the use of NIR-ICG-angiography in comparison to the clinical judgment of a surgeon. The intraoperative use of ICG-angiography usually led to preservation of bowel that would have otherwise been resected, if only traditional clinical methods had been used. Whenever NIR-ICG-angiography revealed a more extensive length of ischemic bowel, the finding was confirmed either through histological examination or through a postoperative course consistent with the ICG findings.

There were, however, two major studies that showcased the limitations of NIR-ICG-angiography in acute settings. Notably, the biggest study included in our review by Joosten et al. [21], was the one that raised the most alarms with its results. The group of patients where the NIR-ICG-angiography made an impact in the surgical management (resulting in either a more conservative or a more aggressive approach), exhibited a higher mortality rate, as well as higher re-operation and advancing ischemia rates in the more conservative group. These results are concerning, considering that the number of re-operations and mortality were secondary endpoints of the study. The authors raised important points regarding the limitations of NIR-ICG-angiography. Specific points mentioned included the low objectiveness of the method, with often no clear cut-off margins where the ischemic bowel becomes necrotic as well as a substantial learning curve, that requires familiarity and experience. Indeed, a paper by Hardy et al. [27], was able to prove a dissonance in the interpretation of bowel perfusion with NIR-ICG-angiography between surgeons of different expertise levels with the method.

We are currently seeing some work being done towards objectifying the interpretation of ICG-based results. Specifically, several studies have recently been published regarding quantifying the images produced with the help of ICG and determining a threshold that defines bowel viability [28], [29], [30]. These studies have demonstrated that several quantifiable parameters (peak fluorescence value, time from ICG injection to the beginning and peak of fluorescence, slope) seem to have a predictive value regarding anastomotic leakage and thus, bowel perfusion. However, as Kong et al. [31] noted in a 2022 review, further studies are still needed to determine the cut-off values that accurately indicate bowel ischemia. Furthermore, Kong’s review points out an issue we also observed in our article: the lack of homogeneity in several parameters like ICG dosage, imaging system and even observation distance. Ahn et al. [32] addressed this problem with clinical and *in vitro* experiments, and concluded that conditional factors cause significant alterations in quantitative values, even proposing a standardised observation distance of 4–5 cm to optimize results.

The second study included in our review that showcased negative results with the use of NIR-ICG-angiography was conducted by Karampinis et al. [18]. In two out of 52 cases, NIR-ICG-angiography revealed adequate bowel perfusion, a fact that was disproved through histological and endoscopic findings. The authors specifically point out that the ischemia shown in both cases was limited to the mucosa, whereas intraoperative observations with ICG are focused on serosal perfusion. This was also demonstrated in an experiment by Seeliger et al. [33], which highlights that only observing perfusion of the serosa might lead to an underestimation of the true length of ischemic bowel.

As demonstrated in our review and several other studies, intraoperative use of NIR-ICG-angiography for real-time estimation of adequate bowel perfusion still has several limitations, that need to be considered when using it. However, we believe that, with some modifications, NIR-ICG-angiography can find its use in emergency surgery.

Future prospects include the use of artificial intelligence, for example machine learning algorithms to analyze quantitative parameters. Several experimental studies have already shown promising results and verify the feasibility of this hypothesis [34], [35], [36].

Further possible solutions to overcoming the boundaries of NIR-ICG-angiography, might include combining it with different methods. An example is a paper by Studier-Fischer et al. [37] that merges NIR-ICG-angiography with a different method of visualization of bowel perfusion, hyperspectral imaging (HSI). Hyperspectral imaging is a method of analyzing tissue spectral response over a spectrum of electromagnetic wavelengths, to provide information regarding tissue oxygenation. An alternative solution involves combining NIR-ICG-angiography with laser speckle contrast imaging (LSCI), a technique that relies on scattering laser particles through tissue in speckle patterns to reveal perfusion [38, 39]. These combined techniques have proven superior to the sole use of each method on its own.

## Summary and outlook

The ability to accurately identify the length of the ischemic bowel in emergency surgery is vital in reducing the rate of anastomotic leakage, reoperation and morbidity. The positive results of the use of NIR-ICG-angiography in an elective surgical setting, raise the subject of whether it can be established as a similarly useful tool in emergency abdominal surgery.

As seen in this review, the studies concerning this question are few and mostly retrospective analyses. Furthermore, the lack of standardization in the ICG dose, methodology and devices used has been noted. Other important characteristics of the examination, such as observation time from injection to peak fluorescence or observation distance, are barely reported. If the method is to be optimized through standardization and quantification, more studies considering these parameters are obligatory.

The results of Joosten [21], showing increased reoperation, mortality and progressive ischemia rates in cases where NIR-ICG-angiography was used in decision-making, are concerning. The authors of the study offer several justified reasons for these results and emphasize the necessity of additional research. The subjective nature of the method, as seen in the variability in user interpretation of the same examination, is being pointed out as a major pitfall. Karampinis et al. [18] raised an additional limitation of visualising serosal vs. mucosal perfusion with the help of NIR-ICG-angiography. Specialized surgeon training, establishing quantitative parameters of the intestinal perfusion with the use of artificial intelligence, as well as homogenization of key method characteristics (such as injection dose, observation time and distance, and hardware used) might hold the key to optimizing the method.

The rest of the papers came to encouraging conclusions, with the application of NIR-ICG-angiography bringing notable advantages to the patients, either in preserving the bowel or preventing leakage by identifying ischemic bowel in cases where macroscopical evaluation failed to do so. At the same time, the method was proven to be safe, quick, and easy to use after proper training of the personnel involved. These characteristics make the application of NIR-ICG-angiography ideal in the setting of emergency intestinal surgery, both open and laparoscopic, as a supportive instrument in bowel perfusion assessment.

It is thus safe to conclude that, even with currently existing limitations, NIR-ICG-angiography can play a great role in the evaluation of bowel perfusion in emergency abdominal surgery. Further research in the form of randomized, prospective trials and standardization of the method is nevertheless essential before establishing this technology as an inarguably valuable tool in the surgical arsenal.
